# Manipulating target size influences perceptions of success when learning a dart-throwing skill but does not impact retention

**DOI:** 10.3389/fpsyg.2015.01378

**Published:** 2015-09-14

**Authors:** Nicole T. Ong, Keith R. Lohse, Nicola J. Hodges

**Affiliations:** ^1^Motor Skills Laboratory, School of Kinesiology, University of British Columbia, Vancouver, BC, Canada; ^2^Motor Learning Laboratory, School of Kinesiology, Auburn University, Auburn, AL, USA

**Keywords:** motor learning, feedback, error-processing, motivation, self-efficacy, success evaluation

## Abstract

Positive feedback or experiences of success during skill acquisition have been shown to benefit motor skill learning. In this study, our aim was to manipulate learners’ success perceptions through a minor adjustment to goal criterion (target size) in a dart-throwing task. Two groups of novice participants practiced throwing at a large (easy) or a small (difficult) target from the same distance. In reference to the origin/center of the target, the practice targets were alike in objective difficulty and indeed participants in both groups were not different in their objective practice performance (i.e., radial error from the center). Although the groups experienced markedly different success rates, with the large target group experiencing more hits and reporting greater confidence (or self-efficacy) than the small target group, these practice effects were not carried into longer-term retention, which was assessed after a 1-week delay. For success perceptions to moderate or benefit motor learning, we argue that unambiguous indicators of positive performance are necessary, especially for tasks where intrinsic feedback about objective error is salient.

## Introduction

Becoming skilled at a task or sport is often the ultimate goal of a participant or athlete when self-initiating participation and practice. Although “skilled” performance may be defined in many different ways, what it requires is the ability to produce a movement (form) or outcome with precision and consistency to a preset level or standard of attainment (e.g., Crossman, 1959; Fitts and Posner, 1967; Adams, 1987). As a learner progresses through practice, a reduction in performance errors is typically experienced and this reduction serves to inform both the learner and their coach about the efficacy of a training method and level of skill and success attained (e.g., Lohse and Hodges, 2015).

Error information is available through intrinsic and extrinsic (or augmented) sources of feedback. Intrinsic sources of feedback are those that are naturally occurring consequences of interaction with the task or skill, such as vision and proprioception. Augmented feedback is an external, supplementary source of information about the task or skill (Schmidt and Lee, 2011; Magill and Anderson, 2012). Much of the research in motor learning, involving manipulations to augmented feedback, has been conducted and interpreted within an information processing (cognitive) framework, based predominantly on theoretical ideas of Adams (1971) and later Schmidt (1975, 2003). According to this framework, augmented feedback is argued to provide error information about performance which impacts the learner’s subsequent attempts in an error negating manner. Feedback could play a positive role or a negative role, depending on how and when it was provided and the learners’ degree of dependency on this information (e.g., Winstein and Schmidt, 1990). For instance, the frequency of augmented feedback has a direct impact on learning, with augmented feedback after every trial in practice resulting in less effective learning (as evidenced in a no-feedback retention test), than a practice with a reduced frequency of feedback (e.g., Weeks and Kordus, 1998). Recently, there has been a shift in thinking about how feedback regarding error works to influence learning. In addition to its information processing/cognitive role, it has been argued that the affective role of error feedback for learning is important and that this has mostly been ignored in studies of motor learning over the past 40 years (e.g., Lewthwaite and Wulf, 2012). There is now evidence that manipulations to the perception of error information impact motor learning and how well performance is retained over a retention interval.

Manipulating the goal-criterion is an intervention which researchers have used to influence perceptions of error feedback and interpretations of success during skill acquisition. In a visuomotor adaptation task, where learners experience a mismatch (angular discrepancy) between their actual hand movements and a virtual cursor trajectory representing their hand movement and learn to adapt to this discrepancy, Trempe et al. (2012) assessed the 5-min and 24-h retention of aiming to hit virtual targets. Of the two groups that completed the 24-h retention, the group that practiced with an easy-goal (i.e., a successful trial if the cursor touched the target) outperformed the difficult-goal group (i.e., successful trial only if the cursor completely covered the target) during retention. This advantage for the easy-goal group was despite the fact that both groups were tested under the difficult-goal criterion in retention. Importantly, the two groups did not differ from each other with respect to objective error during practice. The authors suggested that perceived success was an important contributor to memory consolidation processes, possibly through modulation of arousal-related hormones, such as epinephrine, or the reward-related dopaminergic system (e.g., Brashers-Krug et al., 1996; Shadmehr and Holcomb, 1997; Robertson et al., 2005).

In the study by Trempe et al. (2012), the participants were asked once, at the end of practice, how they perceived their performance (i.e., efficacy ratings) using a 5-point Likert scale. The easy goal groups evaluated their performance as being significantly better than participants in the difficult criterion groups. This supports the authors’ claim that it was perception of success that mediated retention improvements. However, because the participants were not asked again in retention, it is possible that the enhanced feelings of efficacy carried through to retention and potentially impacted performance at the moment of testing in retention in addition to or instead of during the consolidation interval.

Adaptation learning has sometimes been noted as a special case of learning, due to the requirement to adapt motor commands in an altered, often artificial or virtual environment (Krakauer, 2009). In these tasks, the error signal is one that alerts to a mismatch between sensory sources of information (e.g., vision and proprioception) that have been abnormally perturbed. As such, adaptation effects are thought to have limited applicability to other motor learning tasks that require adaptations to errors that are naturally occurring consequences of a normally calibrated sensory system (e.g., throwing a dart too high or too low). Moreover, in adaptation tasks, improvements in responding to the mismatch are often implicitly driven (unconscious) and not necessarily aided by explicit strategies in response to errors (e.g., Mazzoni and Krakauer, 2006). Therefore, it would be important to test the generalizability of these results for tasks where the feedback is veridical, error processing and correction is encouraged and that in general, better typify learning episodes more frequently encountered outside the laboratory.

In the only other study to date where goal-criterion manipulations have been used to influence success perceptions, there was again evidence that these perceptions influenced how well a motor “skill” was retained. Using a coincident-anticipation timing task, participants controlled when they received feedback about objective timing error (the degree and direction of error in ms), with the constraint that it was limited to a third of all practice trials (Chiviacowsky et al., 2012). Participants practiced under one of three conditions; (1) a difficult goal-criterion (an error of 4 ms or less was considered a “good” trial), (2) a less difficult or more viable goal criterion (an error of 30 ms or less was considered a “good” trial), or (3) they were not told what constituted a good trial (control group). In a 24-h retention test and an opposite limb transfer test, participants given the difficult goal were less accurate and more variable than the other groups. Although the groups were not statistically different in practice, the difficult group showed a tendency for greater error and variability, suggestive of immediate effects of success perceptions on performance (that arguably carried through to retention). However, because the statistical differences were located after a retention interval, the authors argued that perceptions of success impacted memory processes occurring in the retention interval (i.e., consolidation). After practice ended, on a confidence scale of 1 (not at all) to 10 (very) that participants could perform the task with errors of less than 50 and 30 ms on the following day, both the less difficult goal and control groups indicated higher self-efficacy (i.e., task-specific confidence) ratings than the difficult goal group. As with Trempe et al. (2012), self-efficacy was probed once only, post-practice.

Though the coincident-anticipation timing task used by Chiviacowsky et al. (2012) was arguably more representative of real-world motor skills than a visuomotor adaptation task, the occlusion of visual stimuli during the response phase and lack of naturally-occurring, intrinsic feedback (in addition to the small percentage of trials when feedback was provided), was likely to have resulted in a heavy dependence on the augmented feedback for error detection and judgments of success. It is highly unlikely that participants were able to rely on internal timing mechanisms (i.e., intrinsic feedback) to accurately judge timing errors to the precision of 50 ms (1/20th of a second) or smaller. Hence, from the literature on goal-related manipulations to success, it is difficult to know how well these findings apply to other tasks where objective error is more salient and the success manipulations are interpreted co-jointly with objective error feedback. Stated in practical terms, is it sufficient to loosen the constraints on “success” to enhance learning, such that the same performance in practice (as a comparison group) is made to look more successful than another? Importantly, participants are not aware that there is a comparison group and whether they are performing worse or better than that group (as positive “self-other” comparisons, or what has been termed “social-comparative feedback,” have been shown to enhance learning, e.g., Lewthwaite and Wulf, 2010; Ávila et al., 2012).

In summary, there is some evidence that techniques which promote perceived success are beneficial to motor consolidation and learning, although the strength of these effects and potential mechanisms are still unclear. In both studies by Trempe et al. (2012) and Chiviacowsky et al. (2012), because objective performance was not different between groups in practice, differences in perceptions of success (and potentially efficacy) were purported as explanations for the learning effects. It is also possible that a perception of higher error leads to a more explicit, strategic mode of control, whereby individuals are consciously trying to control/correct performance from trial to trial (e.g., Maxwell et al., 2001; Poolton et al., 2005; Masters and Poolton, 2012). This may be maladaptive for stabilizing performance (Sherwood, 1988; Schmidt, 1991) or for performance under conditions that promote self-awareness and anxiety (such as retention tests or tests performed under dual-task loads). Indeed, participants with the difficult goal-criterion in the study by Chiviacowsky et al. [2012; and similarly in the Trempe et al. (2012) study] may have been trying to correct errors that could not have been corrected or controlled (e.g., improving on timing errors less than a 20th of a second), which might also have negatively impacted their overall retention if the memory representation was less stable over time.

The aim of the current study was to investigate how perceptions of error (and hence success) during practice of a “real-world” motor task, where feedback is a naturally-occurring consequence of performance, would moderate motor learning and retention. Perceptions of error were manipulated by changing the size of the target area in a dart-throwing task. This is a relatively simple method that could be used in other tasks to enhance perceptions of success, without changing the constraints on performance and keeping the objective error alerting role of feedback integral and essentially constant. Participants practiced dart throwing at either a small or a large target from the same distance. Because the throwing distance is identical for both groups and that aiming to the center of the target (i.e., the bullseye on a dartboard) would be the best strategy for success, we expected that objective practice performance [i.e., radial error (RE) from the center] would be matched across the groups. However, the groups would differ with respect to their subjective interpretation of this information and hence perceptions of success, with more successful target “hits” and increased perceptions of efficacy in the large (easy) target group compared to the small (difficult) target group. Based on past research, we expected that participants throwing to the larger target and hence that experienced more success in practice, would show enhanced performance on a delayed (1-week) retention test than participants throwing to a smaller target (i.e., show improved learning). However, objective error during the practice phase was not expected to be different between the groups.

Self-efficacy perceptions were continually monitored throughout practice and before delayed retention. We also assessed how the manipulation to target size affected explicit knowledge and rules generated by participants about how to perform the skill (i.e., strategic control). If potential benefits associated with greater experience of success in practice is related to a less explicit mode of control (i.e., a more automatic and stable type of control) as suggested by Maxwell et al. (2001; Poolton et al., 2005), then the large target group was expected to report fewer rules/hypotheses about dart-throwing than the small target group. As an additional measure of the type of control adopted when performing the dart-throwing task, we also tested participants under secondary task conditions, where they simultaneously performed the throwing task and a tone-counting task. Again, we expected that the large target group would not experience a performance decrement in this condition, while the small target group was expected to show a decrement, indicative of a more explicit mode of control.

## Materials and Methods

### Participants and Groups

Adult, female, right hand-dominant, novice dart players were recruited via posters and advertisements. All participants were volunteers and gave informed consent before participation (in accordance with ethical procedures of the University). Remuneration of $10 per hour was paid. To ensure that only novice players were included, we verified that participants had not played darts on more than three occasions. We pseudo-randomly assigned to group with the constraint that participants were approximately matched for performance based on a pre-test (i.e., throwing nine darts at a dartboard, from the regulation distance of 237 cm). Participants were assigned to either a large target group (large-T; *n* = 28) or small target group (small-T; *n* = 27)^1^.

### Task and Apparatus

**FIGURE 1:**
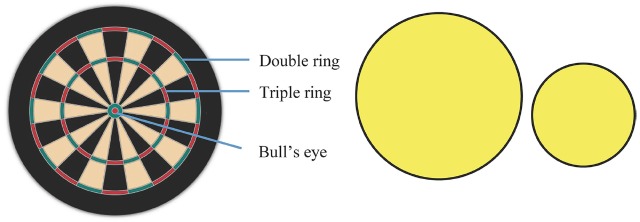
**Schematic of dartboard (at left) and yellow practice targets (large and small).** Bullseye comprised of both the red and green circular zones (the inner and outer bull respectively) in the center of the dartboard.

We modified a regulation size bristle dartboard by removing all metal wire and rings (see Figure 1). The board was then mounted with the center of the bullseye at a height of 1.73 m from the floor. In a pre and post-test, the task was to throw darts at the “bullseye” of the dartboard (defined in this study to comprise of both the inner and outer bull of a standard dartboard). During practice, participants in the large target group and small target group aimed at yellow, circular practice targets of 16 and 7 cm radii, respectively, that were paper targets stuck over the top of a regulation dartboard, with the center of the targets overlaying the origin of the dartboard. The large target was approximately five times larger than the small target in area and covered the whole of the dartboard.

Darts landing outside the yellow targets were recorded as “misses,” scoring no points. “Hits” were hence recorded as darts landing in the yellow target area (see Figure 1). Both hits (checks) and misses (zeros) were recorded by participants on a tally table located to the right of the dartboard in order to increase the salience of success (after every three darts thrown). During the experiment, darts that did not fix on the board were recorded based on later verification by video. Three darts (26 g each) were given to participants to perform the task in sets of three trials. A video camera was set up behind and above the participant so that dart landings of all trials were recorded for subsequent analysis of *x* and *y* coordinate positions in relation to the center of the dartboard (i.e., origin), using Dartfish Prosuite video analysis software (Dartfish, USA)^2^. These values obtained from the video were used for computation of RE (accuracy) and bivariate variable error (BVE; variability) of throwing performance. A standard ruler was used to measure RE during the pre-test, post-test, retention test and secondary task test as a back-up measure of RE (recorded by the experimenter in the interval between each set of three darts). No ruler was used in acquisition as we did not wish to alert participants to errors in relation to the center of the dart-board (rather than merely aiming to hit the yellow target zone).

### Procedure

The experiment took place across two sessions, separated by 1 week (range of 6–8 days). There was a pre-test, followed by a practice (acquisition) phase, then an immediate post-test during the first session and a delayed retention and secondary task test approximately 1 week later. Participants were told not to step over the throw line and not to throw with a sidearm, that is, to keep their arm in the sagittal plane of motion as much as possible (this action was demonstrated). We also told participants that to maximize success at hitting the target a good strategy would be to aim for the center, even though success would be determined based on hitting the target.

Three warm-up throws (which were not recorded) then followed, before participants were introduced to the confidence rating scale which was posted approximately 50 cm to the left of the dartboard (from origin). The scale ranged from 0 to 100% (in increments of 10) and corresponding descriptors were; “0” = “not at all,” “10” = “not sure,” “40” = “somewhat sure,” “70” = “pretty sure,” and “100” = “very sure” (Zimmerman and Kitsantas, 1997). Using the rating scale, participants were prompted for their confidence of landing at least one out of three subsequent throws within each of three pre-determined areas, giving a total of three confidence ratings. The largest of the three pre-determined areas was the area subtended by the “double” ring on the outside rim of a standard dartboard, as shown in Figure 1. This was a size roughly corresponding to the large or easy practice target. The “small” area corresponded to the area subtended by the “triple” ring around the middle of a standard dartboard (roughly corresponding to the small or difficult practice target in size). The smallest pre-determined area (not a practiced target) was the bullseye, which for the experiment consisted of both the inner and outer bull as illustrated in Figure 1.

#### Pre-Test

Before pre-test trial 1, participants indicated a confidence rating for each of the three pre-determined (large, small, and bullseye) areas of the dartboard. Following the ratings, nine pre-test trials ensued.

#### Acquisition

During acquisition, which immediately followed pre-testing, participants were asked to aim at their practice target and were told to make as many “hits” as they could (i.e., land the dart in the yellow target zone). Before Acquisition trials 1, 31, and 61, participants indicated their confidence for making a hit on at least one of the subsequent three trials. A total of 90 darts (across 10 blocks) were thrown during acquisition. One point was awarded for each hit made. In between sets of trials, participants walked over to the tally table to record the hits and misses made in the set while the experimenter removed the darts. The intention for the self-tallying of hits/misses was to enhance perceptions of success (or failure) and to keep them engaged in their practice.

#### Immediate Post-Test

Procedures in the post-test were identical to the pre-test, consisting of nine trials and three confidence probes before the first trial. When trials were finished, participants were interviewed by the experimenter to describe any rule, technique or method pertaining to dart throwing that they had generated or become aware of during practice. These qualitative responses were subsequently categorized and analyzed by the experimenter. The first session ended when participants signed a form confirming receipt of remuneration for participation and agreed that they would not practice or learn more about darts before returning to the laboratory in a week.

After a week, participants returned to the laboratory for Session 2. They first performed three warm-up trials (data not recorded), then administration of the delayed retention and secondary task tests were counterbalanced for order so that half the participants in each group completed one test before the other.

#### Delayed Retention

The delayed retention test was identical to the post-test, with participants performing nine dart throws and indicating their confidence on the three, pre-determined confidence areas, before their first trial.

#### Secondary Task

In addition to the primary task of throwing sets of three darts, participants had to simultaneously monitor and count high pitch tones in an audio sequence. It was verified that participants understood the secondary task in a warm-up tone counting sequence that was similar to the audio sequences played during the test. High and low frequency tones (duration of 300 ms/tone) were interspersed at inter-stimulus intervals between 500 and 1000 ms, to create three random and unique audio sequences. Participants were instructed not to begin their set of three throws until the first tone of the sequence had been played. When participants completed a set of trials, audio playback was stopped, after which participants were prompted to indicate the number of high tones they had mentally counted in the set. In total, three sets of three throws were made in the secondary task condition.

### Data Collection and Analysis

#### Outcome Variables

In addition to recording the number of target hits made during practice as a function of group, a more sensitive measure of outcome accuracy was determined based on RE, which was defined as the absolute distance between dart landing and the origin of the dartboard. Mean RE was calculated for each block of nine trials. Constant error in *x* (horizontal) and *y* (vertical) coordinates of dart landing in relation to the origin were calculated based on post-experiment analysis of the video using Dartfish^©^ Prosuite software. With *x* and *y* coordinates extracted for each trial, RE for acquisition trials was calculated as RE^2^ = (*x*^2^ + *y*^2^). Based on the constant error data, we also calculated BVE (Hancock et al., 1995), based on the following equation:

$$BVE=\sqrt{\frac{1}{k}\sum_{i=1}^{k}{\left(X_{i}-X_{c}\right)}^{2}+{\left(Y_{i}-Y_{c}\right)}^{2}}$$

where: *k* = number of trials

*X_c_* = average constant error on the *X* axis within a test or block

*Y_c_*= average constant error on the *Y* axis within a test or block

Data collected on the first session were analyzed in separate independent *t*-tests on pre-test and post-test group mean RE and BVE. Two other Group (large T, small T) × Block (Blocks 1–10) repeated measures ANOVAs were applied to analyze acquisition mean RE and percentage of target hits, with block as the within-subjects factor. Mean RE and BVE from the second session were analyzed in a Group (large T, small T) × Test (retention, secondary task) repeated measures ANOVA, with group as the between-subjects variable and test as the within-subjects variable.

In the secondary task, we also assessed accuracy of the tone counting response. The responses were either correct or incorrect. We tabulated the number of response errors made by each participant (maximum of three for each individual as there were three tone counting sequences for three sets of dart throws during the secondary test condition). These data were compared across groups using a Mann–Whitney U test.

#### Measure of Self-Efficacy

Our primary measure of self-efficacy was assessed using ratings of confidence. The three confidence ratings provided by the groups during each test phase (i.e., pre-test, post-test, retention test) were analyzed in a 2 Group (large T, small T) × 3 Test (pre-test, post-test, retention test) × 3 Target area (large, small, bullseye) repeated measures ANOVA. The confidence ratings based on the practiced target only, given three times during acquisition, were analyzed in a 2 Group (large T, small T) × 3 Acquisition probe (AQ1, AQ2, AQ3) repeated measures ANOVA.

#### Explicit Knowledge

A comparison of the number of rules or strategies generated following the immediate post-test were analyzed as a function of group using a Mann–Whitney U test.

Partial eta squared (${\text{η}}_{p}^{2}$) values were reported as measures of effect size and *post hoc* analyses were conducted using Tukey’s HSD (*p* < 0.05) for all significant effects. Greenhouse–Geisser corrections were applied for violations to sphericity.

## Results

### Manipulation Checks

#### Target Hits

Confirming the success of the manipulation, the large target group made significantly more target hits (*M* = 7.4/block, SD = 1.4) than the small target group (*M* = 2.9/block, SD = 1.6), *F*(1,53) = 384.17, *p* < 0.001, ${\text{η}}_{p}^{2}$ = 0.88. Although there was also a block main effect, *F*(9,477) = 2.445, *p* = 0.01, ${\text{η}}_{p}^{2}$ = 0.04, indicating a general increase in frequency of hits over the course of acquisition, there was no significant Group × Block interaction, *F* < 1.

#### Self-Efficacy

**FIGURE 2:**
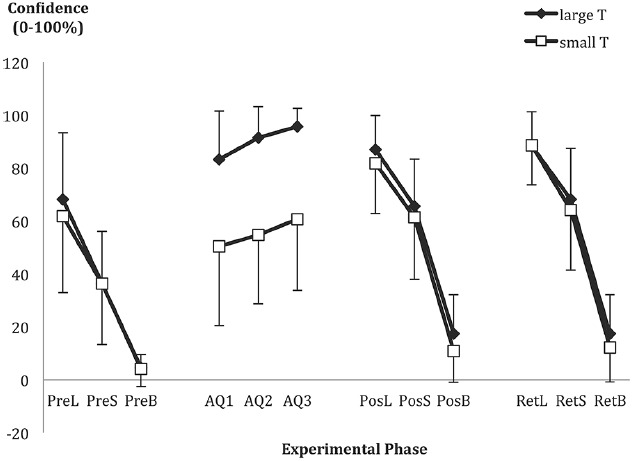
**Mean confidence ratings of hitting at least one out of subsequent three trials for the large (L), small (S), and bullseye (B) pre-determined areas, as indicated before pre-test (PreL, PreS, PreB), post-test (PosL, PosS, PosB), and retention (RetL, RetS, RetB).** Acquisition probes 1–3 were the mean confidence ratings of making a “hit” at least once out of three subsequent trials before the 1st (AQ1), 31st (AQ2), and 61st (AQ3) acquisition trial. Error bars represent standard deviation of the mean.

As shown in the middle of Figure 2, confidence during acquisition increased, *F*(1.7,90.0) = 16.96, *p* < 0.001, ${\text{η}}_{p}^{2}$ = 0.24, with *post hoc* analysis showing a significant increase from AQ1 to AQ3. As would be expected, but serving as a manipulation check, the groups were also different in their confidence in hitting their respective targets, *F*(1,53) = 734.66, *p* < 0.001, ${\text{η}}_{p}^{2}$ = 0.93. The large target group (*M* = 90.1%, SD = 14.1) was more confident about hitting their assigned target than the small target group was about hitting their assigned target (*M* = 55.3%, SD = 27.7). There was no interaction.

### Outcome Measures

#### Mean Radial Error

**FIGURE 3:**
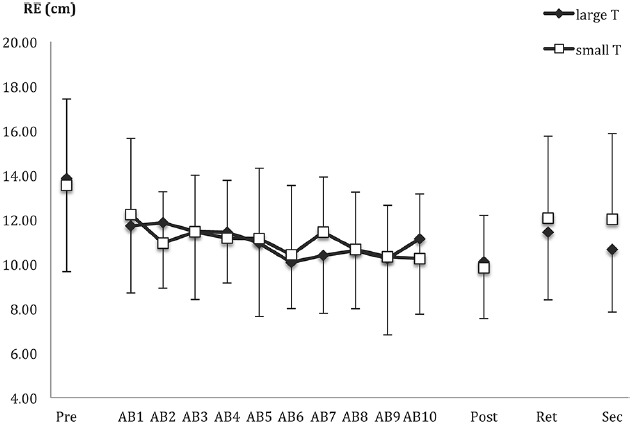
**Radial error (RE) as a function of pre-test (pre), acquisition blocks (AB1-10), post-test (post), retention (ret), secondary task test (sec), and group [large T (target) versus small T].** Data points and error bars represent the mean of nine trials and between-subjects SD.

Outcome accuracy is shown in Figure 3. As expected, the groups did not differ on pre-test accuracy, *t*(53) = 0.28, *p* = 0.78. Despite our predictions, the groups were not significantly different in the post-test, *t*(53) = 0.42, *p* = 0.68, nor was there a significant group main effect, *F*(1,53) = 1.66, *p* = 0.20 or interaction (*F* < 1), when the groups were compared during retention and secondary task tests.

A separate analysis was conducted on the acquisition data. Again, there were no effects involving group (all *F*s < 1), only a main effect of block. This was best described by a linear trend, *F*(1,53) = 11.71, *p* = 0.001, ${\text{η}}_{p}^{2}$ = 0.18, indicating a decrease in RE over the course of acquisition (see Figure 3).

#### Bivariate Variable Error

**FIGURE 4:**
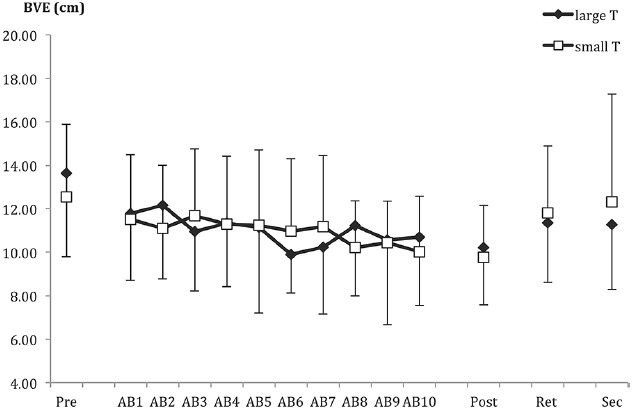
**Bivariate variable error (BVE) as a function of pre-test (pre), acquisition blocks (AB1–10), post-test (post), retention (ret), and secondary task test (sec).** Data points and error bars represent the mean of nine trials and between-subjects SD.

Bivariate variable error is displayed in Figure 4. As with RE, there were no group differences in any of the testing phases (all t and *F* values equal to or <1).

For acquisition, only the main effect of block was significant, *F*(9,387) = 1.96, *p* < 0.05. The groups showed a linear trend of improving consistency (i.e., decreasing BVE) in their throws over the blocks of practice, *p* < 0.01. For all group-related effects, *F*s < 1.2.

#### Accuracy of Secondary Task Tone Counting

The average number of tone counting response errors made by the large target group was 0.7 (SD = 0.9) and 1.0 (SD = 0.8) for the small target group. The groups were not significantly different based on the Mann–Whitney U test, *U* = 294, *Z* = 1.51, *p* = 0.13.

### Process Measures

#### Self-Efficacy Ratings in the Post-Test and Retention

Confidence ratings were assessed before the pre-, post- and retention-tests, for the three dart-board areas (large, small, and bullseye), as displayed in Figure 2. Although there was the expected increase in confidence as target area increased, *F*(1.6,69.2) = 465.35, *p* < 0.001, ${\text{η}}_{p}^{2}$ = 0.92, there were no significant effects involving group (all *F*s < 1.2). There was a test-phase effect, *F*(1.7,72.6) = 51.43, *p* < 0.001, ${\text{η}}_{p}^{2}$ = 0.55, with confidence increasing from pre-test to post-test and into retention, especially for the two practiced targets, as shown by a Test × Target area interaction, *F*(3.2,136.4) = 13.07, *p* < 0.001, ${\text{η}}_{p}^{2}$ = 0.24.

#### Post-Experiment Explicit Knowledge

Neither group reported many rules, strategies or hypotheses for performing the dart-throwing task. The average number reported by the large target group was 2.4 (SD = 1.3); for the small target group this was 2.5 (SD = 0.9). These means were not significantly different, *U* = 357, *Z* = 0.37, *p* = 0.71.

## Discussion

Manipulation of practice target size resulted in greater rates of success for the large target group than the small target group during practice. Self-efficacy, as probed by the confidence ratings, also indicated higher confidence for the large target group than the small target group. These results were expected outcomes of the manipulation, confirming the fact that changes to target size influenced perceptions of success.

Despite the fact that success rates changed across the two groups, this manipulation to target size did not impact learning (cf., Chiviacowsky et al., 2012; Trempe et al., 2012). Either manipulations to target size did not affect the interpretation of error feedback or enhanced perceptions of success in practice do not always translate to improved learning, as assessed by performance in a delayed retention test. There was evidence of improvement during practice for both groups, in that all participants showed a general increase in frequency of target hits, increase in accuracy (RE) and decrease in variability (BVE) over the course of practice. Moreover, the groups did not differ in objective performance error during acquisition even though they differed on subjective rates of success (number of target hits made).

On the impact of perceived success on motor skill learning, the differences in rates of success (due to target hits) did not translate to more permanent differences in self-efficacy as assessed at delayed retention. Although this was not measured in prior work, the absence of group differences in retention for both practiced and non-practiced targets raises issues about the potential of relatively easy target goals to transfer to positive perceptions of efficacy for more difficult goals (i.e., from large to small). Success on a large target does not inform as to success on a smaller target and as evidenced by the confidence scores, the large (easy) target group did not evaluate their chances of success any more favorably than the small (difficult) target group. Perhaps this should not be too surprising, given that going from a difficult to an easier target should result in enhanced perceptions of success, whereas the reverse would result in a decrease. This shows the need to evaluate “success” perceptions with respect to both practiced and non-practiced targets to get a true understanding of whether a manipulation to success is likely to affect learning. In this case, the post-test measures of efficacy were not different between the groups when assessed on the same targets.

Unlike the experimental tasks in Trempe et al. (2012) and Chiviacowsky et al. (2012), the current task presented salient outcome error during skill practice, a source of veridical feedback that may have moderated the success experience intended by the current target goal manipulation. Besides success-related outcome feedback based on target hits and misses, objective performance error (i.e., error in relation to the bullseye/center of target) was available and evident to participants for every dart throw. Hence, this objective source of feedback may have dominated over the subjective success that was assumed to be experienced with target hits. In previous work (i.e., Chiviacowsky et al., 2012; Trempe et al., 2012), because of the lack of objective error information and the greater dependency on augmented feedback during task performance, perceptions of success were likely less ambiguous.

From the goal setting literature, it appears that moderately difficult goals are most motivating for learners (see Atkinson, 1964; Locke, 1968; Bar-Eli et al., 1997) and will also avoid a ceiling that leads to learners setting other (more difficult) personal goals. Participants in the large target group were successful at hitting their target approximately 82% (mean of 7.4 out of maximum 9) of the time during practice. Since they were performing close to the ceiling, it is likely that they had spontaneously set themselves a more difficult personal goal (see Bandura and Simon, 1977) or attributed success to an overly easy task (Bandura, 1998). The saliency of error in relation to the center of the target may have thus muted perceptions of success that potentially contribute to overnight consolidation processes for both groups. With these considerations, manipulation checks for potential personal goals should be in place for future studies using tasks that provide extra sources (or ambiguous sources) of outcome error, to ensure that the participants had accepted the prescribed goal criterion for evaluations of success. What seems to be a conclusion from this emerging research about the affective role of feedback for learning is that moderations to learning via success perceptions only occur when these perceptions are unambiguous. If naturally-occurring objective error is available, even if participants may feel more or less successful on another criterion, this source of information appears to dominate how well a task is learned and hence the influence of success perceptions.

It is also important to point out that the groups did not differ on the secondary task, which was expected to give some indication of explicit control. There was no evidence that during practice the small target group acquired more explicit knowledge (rules and hypotheses) about how to throw the dart (i.e., determining error correction strategies) which are arguably more susceptible to performance breakdowns when working memory demands are increased through the addition of a secondary task load (cf., Maxwell et al., 2001; Poolton et al., 2005; Masters and Poolton, 2012).

The affective role of error feedback in motor learning has garnered and generated much research interest of late (e.g., Lewthwaite and Wulf, 2010; Ávila et al., 2012; Chiviacowsky et al., 2012; Trempe et al., 2012). In this study, we have highlighted limitations to the effectiveness of positive or success-related feedback for learning. Manipulating performance perceptions through different goal-criteria, we showed that whilst target size moderates perceptions of efficacy during practice, the differences in perceptions of success (at least with respect to measures of confidence and successful target hits) did not lead to differences in retention. It appears that when veridical objective error is available, the saliency of this feedback moderates or overwrites subjective success manipulations intended to benefit learning. It is also possible that goals that are too easy are not evaluated positively or judged as motivating or rewarding (e.g., Caplin and Dean, 2008). In comparison to Trempe et al. (2012), success was achieved on 62% of trials for the easy-target group in their study, whereas this was 82% in the current study. Similarly, in Chiviacowsky et al. (2012), the less difficult goal group was “successful” on 53% of their feedback trials. Given these differences in success, it is possible that there are limits to which “success,” associated with achieving goal-criteria, would actually translate to positive perceptions of performance for the learner. In comparison to the relative success of social comparative feedback (i.e., superior performance relative to peers) in influencing perceptions of success (e.g., Ávila et al., 2012), manipulations to the target goal rely on a subjective perception of what the feedback means. Therefore, feedback is likely to be less effective at changing perceptions of success and subsequently learning, when the goal-criterion is easily attained, when other sources of performance feedback are available and when comparisons are not made to other people.

Regardless of the reason for the lack of positive effects on learning associated with easier target goals, we would argue that caution is needed when using goal criteria to manipulate success perceptions and learning. In order to tap into potential affective gains associated with positive or success-related feedback, instead learners might benefit more from receiving positive social comparative feedback or evaluating their performance on unambiguous standards/goals when objective error feedback is present.

### Conflict of Interest Statement

The authors declare that the research was conducted in the absence of any commercial or financial relationships that could be construed as a potential conflict of interest.

## Footnotes

1 Two participants were excluded from analyses as their mean pre-test radial error scores were more than three SDs greater than the mean pre-test radial error of the other participants.

2 For the first five participants assigned to each group, videos were only recorded of dart landings during the acquisition phase. Hence, *x* and *y* coordinates, and bivariate variable error were not computed for the test phases of these 10 participants (only radial error was recorded from measurements made with a ruler at the time of testing).
